# Impacto de la arquitectura del hospital en la experiencia de parto: un estudio fenomenológico con madres expertas en su diseño

**DOI:** 10.23938/ASSN.1059

**Published:** 2024-02-13

**Authors:** Laura Cambra-Rufino, Angela E. Müller, Marta Parra Casado, Azucena Pedraz Marcos

**Affiliations:** 1 Universidad Politécnica de Madrid Escuela Técnica Superior de Arquitectura Departamento de Construcción y Tecnologías Arquitectónicas Madrid España; 2 Parra-Müller Madrid España; 3 Virai Arquitectos Madrid España; 4 Instituto de Salud Carlos III Unidad de investigación en cuidados y servicios de salud (Investén) Madrid España

**Keywords:** Investigación Cualitativa, Salas de Parto, Entorno del Parto, Diseño de Instalaciones Basado en Evidencias, Arquitectura y Construcción de Hospitales, Qualitative Research, Delivery Rooms, Birth setting, Evidence-Based Facility Design, Hospital Design and Construction

## Abstract

**Fundamento:**

El lugar donde paren las madres condiciona su proceso de parto y nivel de satisfacción. El objetivo de este estudio es identificar las experiencias y percepciones acerca de los elementos de diseño del entorno del parto hasta el alta hospitalaria, que influyen en la experiencia de las madres a largo plazo.

**Metodología:**

Investigación fenomenológica de tipo método biográfico, a través del análisis temático inductivo de veinticinco testimonios de parto en el hospital, escritos por madres arquitectas, ingenieras, paisajistas o diseñadoras de interiores.

**Resultados:**

Los resultados se organizaron en cuatro temas y siete subtemas. El primer tema es la “Impresión a primera vista y largo plazo” que se subdivide en los subtemas “Itinerario despersonalizado en accesos y pasillos” y “Búsqueda instintiva de conexión con la naturaleza”. El segundo tema trata sobre el “Acompañamiento y arropamiento durante el proceso de parto” y se subdivide en “*Como en un hotel*: espacio para el movimiento y adaptación personalizada” y “*Desamparo, frío e incertidumbre*: espacios donde estar contra su voluntad”. El tercer tema son los “Daños (en espacios) colaterales”, que engloba “La integración de los aseos en el proceso de parto”, los “Quirófanos inmutables ante el parto por cesárea” y las “Salas de neonatos que no integran a las familias”. El cuarto tema incluye “Propuestas de mejora para nuevos diseños”.

**Conclusiones:**

Esta investigación permite profundizar en aspectos de diseño identificados en literatura reciente y mostrar que son necesarios más estudios que incorporen la experiencia de la mujer en el proceso del parto para promocionar políticas de diseño basadas en evidencias.

## Abstract (en)

**Background:**

The birthplace has a crucial role in shaping the childbirth experience and mothers’ satisfaction levels. This study aimed to identify the experiences and perceptions that may have an impact in the long-term on mothers’ birthing experience, considering hospital design features in the birthing environment until discharge.

**Methods:**

Inductive thematic analysis of twenty-five hospital labor testimonies employing a phenomenological research approach and utilizing a biographical method. Participants were women with a professional background in architecture, landscape architecture, engineering, or interior design.

**Results:**

The results are organized into four themes and seven subthemes. The first theme is “First sight and long term impression” which is subdivided into the subthemes “Depersonalized itinerary in entrances and corridors” and “Instinctive search for connection with nature”. The second theme deals with “Accompaniment and tucking in during the birthing process”, subdivided into “*Hotel-like*: space for movement and personalized adaptation” and “*Helplessness, cold and uncertainty: spaces* to be against one’s will”. The third theme is “Damage in collateral rooms”, which includes “The integration of toilets in the birthing process”, “Operating rooms unchangeable in the face of cesarean delivery” and “Neonatal units that do not integrate families”. Finally, the fourth theme includes “Improvement proposals for new designs”.

**Conclusions:**

This study contributes to the existing literature by deepening the understanding of the design features identified in hospitals in recent studies. Further research incorporating the experiences of women in the birthing process is needed to facilitate evidence-based design policies.

## INTRODUCCIÓN

El proceso de parto en la mujer es un momento transformador y decisivo que afecta a la calidad de vida del recién nacido, pero también al nivel de salud, económico y social de la nueva familia^1^^,^^2^. El parto condiciona la experiencia de la nueva maternidad tanto por su carácter íntimo, físico, psicológico y emocional, como por la influencia de su entorno social, ambiental, organizacional, y político^3^.

La literatura revisada incluye estudios que utilizan distintas técnicas cualitativas -como la observación, los vídeos, las entrevistas o los grupos focales- para explorar la experiencia de parto de la madre^4^^-^^6^, así como estudios cualitativos que abordan el fenómeno desde el punto de vista de las matronas^7^^-^^9^ e incluso de las personas acompañantes^10^.

Una parte fundamental de la experiencia de la madre viene condicionada por el lugar del parto, que puede ayudar o dificultar el proceso fisiológico e influye en su nivel de satisfacción^4^^,^^11^. Existe un volumen creciente de literatura científica que demuestra la influencia del entorno físico del hospital en los resultados sanitarios^12^^-^^14^. Respecto al bloque obstétrico, varios estudios relacionan elementos de diseño como la bañera obstétrica con la reducción de las tasas de episiotomía, los partos instrumentales y las cesáreas^15^^,^^16^.

A nivel nacional, los estándares y recomendaciones^17^ incluyen aspectos relacionados con su estructura y recursos materiales. Otros países como Australia llevan años desarrollando una herramienta que facilite la implantación de los avances en investigación en cuanto al diseño del entorno del parto en el hospital^18^^-^^21^.

En un reciente estudio sueco^22^ se ha comprobado que poder ajustar la sala donde las madres paren en el hospital para adaptarla a sus necesidades (como regular la iluminación natural y artificial, modificar la posición de la cama, parir en vertical aprovechando la gravedad, usar una bañera de partos, escuchar sonidos naturales, o tener una decoración doméstica con dispositivos clínicos ocultos), hace que la experiencia de parto recordada hasta un año después sea más positiva comparada con la experiencia en una sala de partos tradicional. A pesar de que este estudio se basa en datos cuantitativos del mayor nivel de evidencia, obtenidos a partir de un ensayo controlado aleatorizado donde utilizan el cuestionario validado CEQ2^23^^-^^24^, sus conclusiones recomiendan combinar sus resultados cuantitativos con estudios cualitativos que exploren en profundidad los elementos de diseño que afectan a la experiencia de la madre y perduran en su recuerdo. Sin embargo, no se ha encontrado ningún estudio que explore el impacto del diseño arquitectónico en la experiencia de parto en el hospital a largo plazo, entendido como el periodo posterior a 12 meses después del parto.

Por este motivo, el objetivo de esta investigación es identificar las experiencias y percepciones acerca de los elementos de diseño del entorno del parto hasta el alta hospitalaria, que influyen en la experiencia de la madre a largo plazo.

## MATERIAL Y MÉTODOS

Estudio cualitativo exploratorio de testimonios de partos relatados por madres que, por su formación y profesión, tenían la capacidad de profundizar sobre el diseño arquitectónico del entorno del parto (como arquitectas, ingenieras, paisajistas o diseñadoras de interiores). El periodo de recepción de testimonios fue de septiembre de 2019 a enero de 2022.

### Diseño

El marco teórico sobre el que basamos la investigación fue el fenomenológico, cuyo objetivo es comprender un fenómeno determinado a partir de las distintas experiencias de las personas que participan en él^25^, usando el método biográfico para acercarnos a la narración de estas experiencias^26^^,^^27^. El estudio consistió en el análisis cualitativo exploratorio de testimonios de partos.

### Participantes

La población de estudio fueron las madres arquitectas, ingenieras, diseñadoras o paisajistas, mayores de 18 años que hubieran tenido una experiencia de parto en un hospital. Se excluyeron aquellos testimonios que describían partos fuera del ámbito hospitalario.

El muestreo fue por propósito^28^, con la estrategia de contar con las personas más adecuadas para dar cuenta del objeto de estudio, teniendo en cuenta la tipicidad de las participantes y la necesidad de constatar la influencia del entorno en la vivencia del parto. Se complementó por un muestreo en bola de nieve de manera informal, al transmitir el llamamiento de unas participantes a otras. El tamaño muestral estuvo condicionado, finalmente, por el tiempo asignado para la recogida y la saturación de los datos.

### Captación y recogida de datos

**Figura 1:**
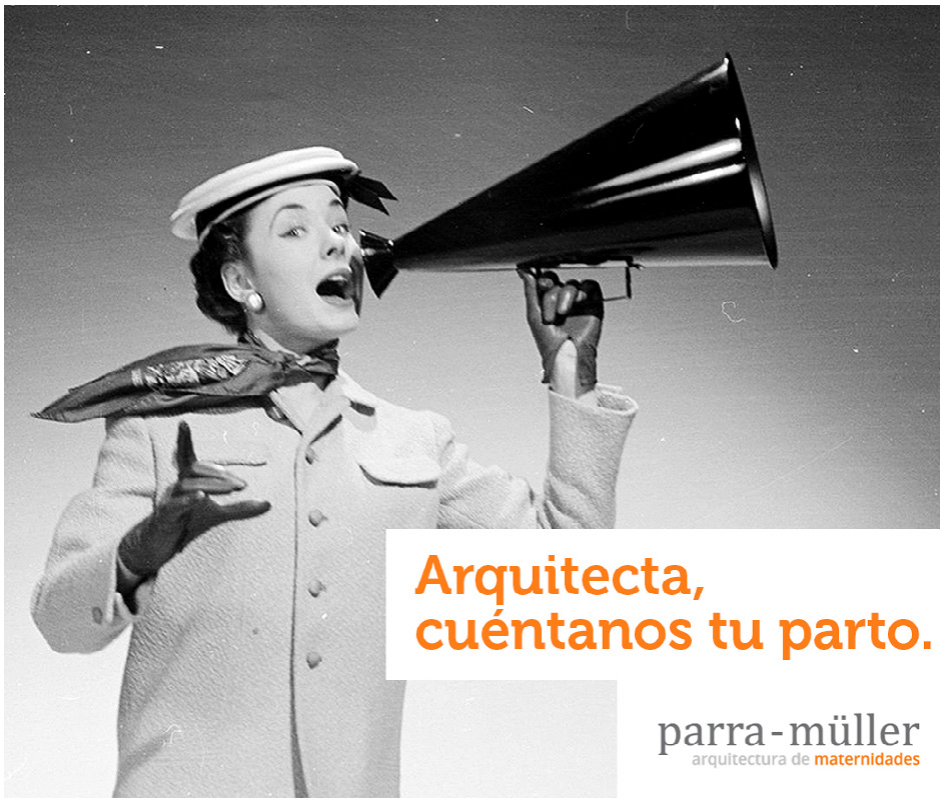
*Flyer* del llamamiento difundido en la página web http://www.arquitecturadematernidades.es

El estudio se organizó en tres fases: en primer lugar, el llamamiento a la participación; en segundo lugar, la recogida de los testimonios; y una tercera fase de análisis. El llamamiento consistió en un *flyer* (Fig. 1) en el que se informaba del marco que fundamentaba el estudio, la humanización del parto, y el objetivo del estudio, y en el que se pedía a las madres que compartieran su relato de parto para poder visibilizar el impacto de la arquitectura, sin límite de extensión o de formato. Esta información se divulgó en varios idiomas (castellano, inglés y alemán) por canales como: correo electrónico; un post en la página web del estudio de arquitectura Parra-Müller; otro post en la página de la misma organización en *Facebook*; y otro en los perfiles de *LinkedIn* de dos autoras (AM y MP).

Durante la recogida de datos, las madres enviaron su relato de distintas formas: contestando al mail que habían recibido; a través del correo electrónico proporcionado en el *blog*; o por mensaje directo desde las redes sociales (*Facebook*, *LinkedIn* o *WhatsApp*).

### Análisis de datos

Se aplicó el análisis temático inductivo (con ayuda de la herramienta *Atlas.ti*) y según los pasos propuestos por Braun y Clarke^29^: familiarización con los datos, generación de códigos iniciales, búsqueda, revisión y definición de temas, y su contextualización con la pregunta de investigación. El proceso comenzó con un análisis temático inductivo de los testimonios, a partir de la lectura y relectura de los mismos. A través de este proceso se atribuyeron códigos iniciales a los datos, sobre cuya reflexión surgieron los temas principales. Los temas fueron entonces revisados por todas las investigadoras, nombrados e incluidos en un informe junto con los subtemas y citas.

### Consideraciones éticas y de calidad

La información proporcionada por las madres fue tratada de acuerdo con la Ley de Protección de Datos Personales y garantía de los derechos digitales^30^. Este estudio no requirió la aprobación por un Comité de Ética, y todas las participantes firmaron un documento de consentimiento informado. Para preservar el anonimato, se asignó a las participantes un número de estudio y todos los nombres se eliminaron del análisis y de los informes escritos. Para mejorar el rigor metodológico, se siguieron los criterios de calidad en investigación cualitativa de Lincoln y Guba^31^. Para garantizar la credibilidad, los datos se presentan como citas textuales y se explican según la interpretación de las investigadoras para ilustrar la riqueza de los datos. Las investigadoras utilizaron la reflexividad durante toda la investigación, analizando su propia posición sobre el tema para garantizar la confirmabilidad de los datos. La transferibilidad se aplicó mediante muestreo por propósito y saturación de datos^31^.

## RESULTADOS

Se recibieron 41 testimonios, de los que se seleccionaron 25 (61%) elaborados por 24 madres (una de ellas describió el parto de sus dos hijos) (Tabla 1). Se desestimaron 16 testimonios que no hacían referencia al objetivo del estudio (n=5) o que resultaban redundantes (n=11).

**Tabla 1:** Características de los partos narrados por las participantes

Parto	Orden	Tiempo*	Tipo	Tipo de hospital	Ciudad
1	1º	2	Vaginal	Público	Huesca
2	1º	-	Cesárea intraparto	Público	Londres
3	-	-	-	-	-
4	2º	0,17	Cesárea programada	Privado	Valencia
5	1º	1,25	Cesárea programada	-	Barcelona
6	1º	23	Vaginal	Privado	Marbella
7	1º	1	Cesárea programada	Privado	Madrid
8	2º	-	Vaginal instrumentado	-	Madrid
9	1º	0	Vaginal	Público	Palma de Mallorca
P10	1º	20	Vaginal instrumentado	Privado	Madrid
P11	1º	0	Vaginal	Público	Leipzig
P12	1º	12	Cesárea programada	Privado	Madrid
P13	2º	8	Vaginal	Público	Madrid
P14	2º	14	Cesárea programada	-	Barcelona
P15	2º	2	Vaginal	Privado	Madrid
P16	-	-	-	-	Colombia
P17	2º	0	Vaginal	Público	Madrid
P18	1º	-	Vaginal	Público	Torrejón de Ardoz
P19	1º	2	Vaginal	Público	Palermo
P20	-	13	-	-	-
P21	1º	1	Vaginal	Privado	Madrid
P22	1º	14	Vaginal instrumentado	Privado	Almería
P23	2º	-	Vaginal	Público	Holanda
P24	1º	1	-	Privado	-
P25	-	8	Cesárea programada	-	Barcelona

*: años transcurridos desde el parto hasta el momento de ser narrado; -: sin datos. Los partos 12 y 13 corresponden a la misma madre.

La mayoría de las madres del estudio eran primíparas (n=14; 56%), mientras que el 28% (n=7) describió el parto de su segundo hijo, y del 16% (n=4) no se obtuvieron datos. Los partos ocurrieron principalmente en hospitales españoles (n=17; 68%), con la misma proporción de hospitales públicos y privados, y con más de la mitad por parto vaginal o vaginal instrumentado (n=14; 56%).

Del análisis inductivo de los testimonios, agrupamos los resultados en cuatro temas y siete subtemas, tal y como se describen a continuación (Tabla 2).

**Tabla 2:** Relación de temas y subtemas

1. Impresión a primera vista y largo plazo
1.1. Itinerario despersonalizado en acceso y pasillos
1.2. Búsqueda instintiva de conexión con la naturaleza
2. Acompañamiento y arropamiento durante el proceso de parto
2.1. *Como en un hotel*: espacio para el movimiento y adaptación personalizada
2.2. *Desamparo, frío e incertidumbre*: espacios donde estar contra su voluntad
3. Daños (en espacios) colaterales
3.1. La integración de los aseos en el proceso de parto
3.2. Quirófanos inmutables ante el parto por cesárea
3.3. Salas de neonatos que no integran a las familias
4. Propuestas de mejora para nuevos diseños

Tema 1. Impresión a primera vista y largo plazo

En este tema se incluyeron aspectos que influían tanto en la primera impresión del hospital como aquellos que quedaban grabados en el recuerdo de las madres.

Subtema 1.1. *Itinerario despersonalizado en acceso y pasillos*

En algunos discursos, las madres identificaban la primera impresión que les producía el hospital como un aspecto determinante para decidir dónde querían parir.

*“Entramos por urgencias, no hay nadie esperando, me reciben con una silla de ruedas, directa a correas. En una sala que no sé lo que es (no es sala de espera, no es habitación, no es sala de partos) (…) Se van y allí me quedo yo mirando la sala sin saber dónde estoy. (…) Disculpen, me voy, no quiero parir aquí.” P1*

Un factor que provocaba insatisfacción en las madres eran los sucesivos traslados entre salas distintas que les hacían sentir como *el carro de la compra* o como *mujer objeto*. Asociaban la rotación por múltiples espacios con la rotación entre los distintos facultativos que les atendían, lo que daba la sensación de que nadie se hacía cargo del proceso.

*“En el paso por las distintas salas me siento como un jamón en un secadero industrial, trozo de carne pasando por instalaciones que me son totalmente ajenas dentro de la cadena de producción de los partos.” P22*

No solo percibían despersonalización en el proceso de parto, sino también en las distancias entre la puerta de entrada al hospital y la ubicación de los paritorios, que en ocasiones estaban ubicados en la planta sótano.

*“…odio profundamente que los paritorios estén en sótanos. ¿Por qué? Cuando te llevan en esa camilla desde la habitación hasta el paritorio......a través de pasillos......ascensores que bajan mil plantas.” P4*

Subtema 1.2. *Búsqueda instintiva de conexión con la naturaleza*

La conexión con la naturaleza aparecía en todas las fases del proceso del parto. Para disminuir la ansiedad que sufrían en algunos casos e intentar evadirse, las madres procuraban mirar hacia el exterior del edificio lo que solamente era posible si había ventanas y si sus vistas eran agradables.

*“Recuerdo que cuando conseguí andar un poquito y pasearme para ejercitar lo único que quería era ver la calle y le pregunté a una enfermera dónde había una ventana que se viera la calle y me dijo ‘no hay, lo sé, es horrible’.” P2*

**Figura 2:**
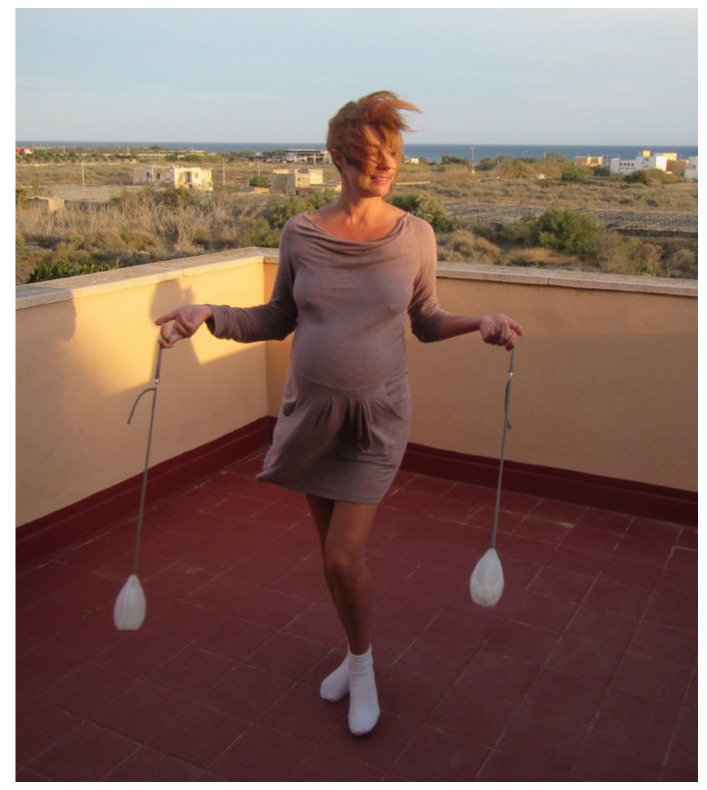
Dilatación en terraza. [Se dispone de consentimiento informado firmado para publicar fotografía con rostro visible].

La luz natural y la vegetación mantenían un papel protagonista en el recuerdo de buenas experiencias. Las ventanas funcionaban como un distractor positivo cuando tenían vistas a espacios naturales (el jardín, la palmera o el mar). También valoraban positivamente poder dilatar en una terraza del hospital (Fig. 2).

*“Otro recuerdo que guardo precioso es cuando mi hijo vino a verme con mi madre. Tenía unas ganas tremendas de verle. Me hice la fuerte y salimos a caminar al pasillo. Los dos recordamos ese momento (él era muy pequeño). Recuerdo el corredor lleno de luz, con cristaleras dando al jardín.” P8*

*“Bailo las cariocas en la terraza mirando al mar y pensando en cómo va a ser mi hija (…) me encanta la sensación de salir al aire libre de la terraza con el sol y el viento dándome en la cara, me hace sentir poderosa y unida al mundo.” P22*

Tema 2. Acompañamiento y arropamiento durante el proceso de parto

Subtema 2.1. *“Como en un hotel”: espacio para el movimiento y adaptación personalizada*

Los elementos estructurales de la habitación, la disposición del mobiliario y los elementos ambientales, contribuyeron también a proporcionar una experiencia agradable en la estancia. Entendimos por habitaciones todas las salas donde permanecía la madre durante más tiempo hasta el alta hospitalaria, ya fuera en un bloque obstétrico secuencial (con habitaciones separadas para la dilatación, parto y recuperación), como en el integrado (con una unidad de trabajo de parto, parto y recuperación), o en la habitación de hospitalización. Los comentarios positivos enfatizaban la amplitud, la tranquilidad, el mobiliario para acompañantes, la posibilidad de moverse libremente y la iluminación natural.

*“Llegamos a la habitación. Es una habitación individual, con baño, luminosa, con espacio, acogedora. Me siento en un hotel, no en un laboratorio. Me siento persona, no número de barras. En la habitación se respira tranquilidad, cariño.” P1*

Se valoraba positivamente poder escuchar música propia o audios de hipnoparto. Un aspecto importante era preservar la intimidad de la madre en su parto, que se resolvía adecuadamente cuando se utilizaban vidrios en las ventanas sin visión desde el exterior.

*“Otra pared estaba ocupada con un ventanal enorme que ya se dio prisa la matrona en explicarme que, aunque yo viera todo con claridad desde dentro, desde fuera no podían ver nada del interior.” P11*

En relación a la cama de partos se apreciaba tener la posibilidad de adoptar distintas posturas, y contar con otro tipo de mobiliario, como taburetes, que facilitaran el parto en vertical. Por último, valoraban positivamente el estímulo agradable de distintos sentidos: la vista, a través de cuadros artísticos; el olor, con geles aromáticos; el tacto, con texturas suaves en las cortinas; o incluso el gusto, cuando se les ofrecía chicles de sabores.

*“Una cama que me permitía muchísimas posturas, unas matronas simpatiquísimas (súper formadas) que no solo me recomendaban posiciones para dilatar mejor, sino que además me trataban como si fuera una amiga de toda la vida. En el ordenador con la monitorización nos dejaron poner la música que nos apetecía. Recuerdo que una matrona me ofreció un chicle, ¡cómo lo agradecí! sabor a fresa fresquita... y un gel de manos con olor a moras. Una experiencia para los sentidos que, con la subida de oxitocina natural, acompañó a tener una experiencia inolvidablemente bonita.” P9*

Subtema 2.2. *“Desamparo, frío e incertidumbre”: espacios donde estar contra su voluntad*

Por el contrario, otras experiencias hablaban de espacios que no favorecían el acompañamiento o el deseo de permanecer en ellos. La sensación que transmitía la habitación podía ser negativa cuando la superficie era muy reducida y no había espacio para moverse, ni para objetos personales, o para acomodar a los acompañantes. También disgustaba el mobiliario austero, la iluminación artificial directa, que no se podía regular, o que era demasiado blanca. Los comentarios acerca de la inadecuada climatización fueron un factor redundante en los testimonios, debido a la incapacidad de controlar la temperatura y los bruscos cambios que sufrían las madres, que en su mayoría manifestaban pasar frío. En ocasiones, además, se le sumaban los olores espesos y la falta de ventilación.

*“Pero no podía parar de tiritar, casi eran estertores, estaba helada, posiblemente la temperatura era baja y se sumaba el agotamiento y el esfuerzo sobrehumano que había pasado.” P10*

*“Lo peor es en sí el paritorio, verde, desnudo, con una silla horrible como de dentista, sin ventanas y con una luz dándote en la cara, hacía calor y como que no ventilaba y con un olor fuerte y espeso. Además, en uno de los partos el brazo de la silla estaba roto y ni siquiera servía de ayuda para, agarrarte y empujar...era incomodísimo.” P6*

La acústica era otro aspecto relevante por las molestias que producían el control de enfermería y las constantes visitas. En cuanto a la intimidad, a algunas madres les llamaba la atención cuando se recurría a sistemas improvisados como el papel de mantel para oscurecer las ventanas de las puertas. También les molestaba la posición relativa de la cama con respecto a la puerta para evitar su visión desde el pasillo.

*“… la cama como casi siempre en los hospitales estaba en la mitad de la habitación (sin ninguna intimidad), el cabezal orientado hacia la pared y con las piernas hacia el centro de la habitación, al final la puerta.” P23*

Tema 3. Daños (en espacios) colaterales

En este tema se incluyeron espacios clave en el proceso del parto, distintos del paritorio, como el aseo, el quirófano o la sala de neonatos. En estos lugares, las madres identificaron experiencias principalmente negativas, por no responder a las necesidades derivadas del proceso de parto.

Subtema 3.1 *La integración de los aseos en el proceso de parto*

Otros aspectos que producían insatisfacción en las participantes, tal y como reflejan los testimonios, fueron la ubicación del aseo fuera del paritorio, los materiales de acabados fríos al tacto y que producían eco, la iluminación artificial demasiado blanca, los inodoros poco accesibles tras la cesárea y el uso indebido del aseo para almacenamiento de material de limpieza.

*“Para llegar al aseo había que cruzar un pasillo. Estaba con contracciones fuertes y tenía que sujetarme la tripa. En todo esto fue imposible cogerme la bata del hospital, así que se me veía entera, desnuda, por detrás. En el mismo pasillo había una familia al completo de otra parturienta, y me estaban viendo todos mientras yo iba, como podía, al baño: descalza, ¡medio desnuda y gimiendo entre contracciones me arrastraba al baño… ese momento no me gustó mucho!” P20*

Por el contrario, la bañera o una ducha de agua caliente resultaba muy adecuada durante la dilatación. Algunas madres utilizaban el aseo para refugiarse en un entorno más controlado donde sobrellevar el dolor.

*“La ducha caliente era lo único que conseguía distraerme un poco. Para sobrepasar el dolor de cada contracción hice uso de mi imaginación; la pared del baño estaba alicatada, así que me imaginaba una gráfica que representaba mi dolor, como si fuera subiendo por los encuentros entre las baldosas, cuando llegaba al punto de máximo dolor, mis ojos se fijaban en el encuentro entre la pared y el techo... poco a poco hacía un recorrido de bajada por los azulejos tomando conciencia de cómo el dolor de la contracción desaparecía.” P9*

Subtema 3.2 *Quirófanos inmutables ante el parto por cesárea*

Con respecto a los quirófanos, las madres tan solo resaltaron aspectos que aumentaban su insatisfacción. Entre ellos estaba el frío, la luz artificial directa, los sonidos desagradables, la ausencia de ventanas, la incomodidad de la camilla, la separación física y emocional que les producía la tela verde sobre su tripa o los reflejos no deseados que mostraban lo que les estaban haciendo a sus tripas.

*“Aquí viene mi recuerdo más frio… me pusieron una tela verde que no permitía ver el nacimiento y le pidieron a mi marido que esperara fuera. El parto se convirtió en dos espacios, yo a un lado y los doctores al otro hablando de sus cosas, yo no sentía nada.” P14*

*“Me fijé en el techo, había una luminaria redonda que reflejaba un poco lo que estaba pasando al otro lado, empecé mirando, pero en cuanto medio vi lo que parecía mi tripa abriéndose, con el ruido de aparatos y el olor...dejé de mirar.” P7*

Subtema 3.3 *Salas de neonatos que no integran a las familias*

En cuanto a las unidades neonatales, de nuevo todos los aspectos de diseño que recordaban las madres eran negativos. Estos incluían la ubicación de la unidad de neonatología alejada de la hospitalización materna, lo que conllevaba la separación madre-bebé y la dificultad de amamantar. Las ventanas también resultaban relevantes, tanto por no tener vistas al exterior que las distrajeran, como cuando se ocultaban para impedir la visión de la sala de incubadoras desde el pasillo y potenciaban su sensación de desconexión con el bebé. En cuanto al mobiliario y equipamientos para familiares, en todos los casos resultaban insuficientes para propiciar su presencia.

*“Dormí una semana en sillones y echaba mucho de menos una cama para poder recuperar fuerzas después del parto y de la angustia en el hospital. Para poder ducharse los acompañantes había una sola ducha a compartir, lejos de las habitaciones de los bebés. Para desayunar o comer había que salir a la cafetería del hospital y organizar con familiares o amigos turnos para no dejar al bebé sin atención.” P13*

Tema 4. Propuestas de mejora para nuevos diseños

En sus testimonios, las madres propusieron distintas ideas de mejora como: instalar una luz tenue en la habitación para que no les despertara el personal de enfermería por la noche; usar velas LED o una lámpara de luz indirecta regulable; incorporar mecanismos para poder controlar la temperatura; colocar asientos junto a ventanales en los pasillos; contar con un espacio exterior accesible para poder pasear; diseñar los falsos techos (en habitaciones y en pasillos) pensando en su visión desde la cama; tener un microondas en el paritorio para calentar los saquitos de semillas; disponer de almohadas de lactancia; colocar barras de agarre y alzadores en el inodoro del aseo para después de la cesárea; y por último instalar hilo musical y utilizar colores cálidos en los quirófanos.

## DISCUSIÓN

**Figura 3:**
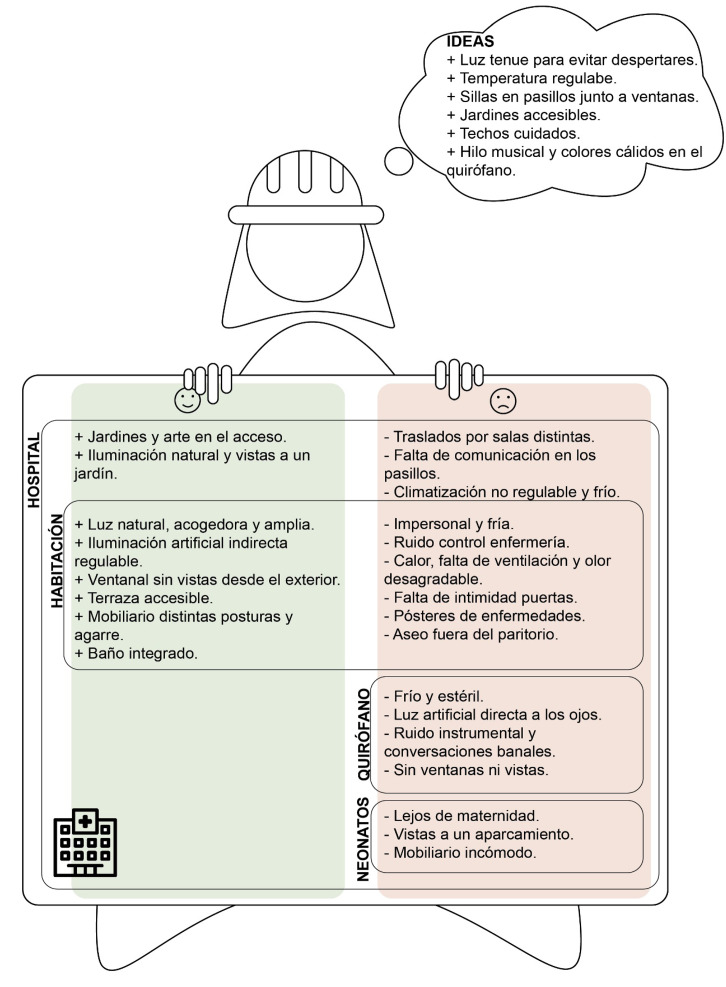
Resumen visual sobre los elementos de diseño que producen experiencias positivas y negativas identificados por las madres.

Los testimonios de madres arquitectas, ingenieras, diseñadoras de interiores o paisajistas nos han permitido explorar de manera detallada determinados elementos de diseño del entorno del parto en el hospital, previamente enumerados en otros estudios. A modo de resumen, las madres identifican elementos de diseño que pueden producir satisfacción o insatisfacción en el hospital, en la habitación, en el quirófano y en la sala de neonatos, además de ideas de mejora generales (Fig. 3).

Una metasíntesis de la literatura enfocada en la experiencia psicológica del parto fisiológico identifica el potencial del entorno físico para favorecer el sentimiento de empoderamiento de la mujer sobre su propio parto^32^. Este instinto biológico de preparar el nido donde va a nacer el bebé^22^ se refleja en los resultados obtenidos, ya que manifiestan la necesidad de adaptar el entorno a unas necesidades agudas muy específicas. Además, se repiten elementos de diseño previamente identificados como facilitadores de una experiencia de parto más positiva^11^^,^^22^ como son el cuarto de baño integrado, la iluminación natural o las texturas naturales. El valor añadido de este estudio reside en su capacidad para profundizar en mayor nivel de detalle y ampliar el abanico gracias a su naturaleza cualitativa y exploratoria. La calidad del sueño y el impacto de las interrupciones por el personal de enfermería o el ruido también se han identificado, aunque en otro tipo de unidades del hospital^33^. Al mismo tiempo, resulta interesante destacar que la vinculación con el espacio exterior durante el proceso del parto es un aspecto detectado en la literatura existente^34^^,^^35^. Por otro lado, la capacidad del diseño de las unidades neonatales en facilitar la implicación de los familiares es un campo de estudio actual^36^.

En cuanto a la propuesta metodológica hay que señalar que, cada vez con más frecuencia, se utiliza la perspectiva de la madre como instrumento para evaluar los cuidados que recibe y mejorar los servicios de atención materna^37^. El interés de este estudio reside en su perspectiva doble: por un lado, obtener el punto de vista de la mujer como madre y, por otro lado, el de la mujer capacitada (por su formación y/o profesión) en definir el entorno hospitalario donde se produce el parto. Resulta imprescindible entender la manera en que las mujeres experimentan los lugares donde dan a luz para poder diseñarlos en base a su experiencia^37^. Si bien no se muestra en las citas del artículo, los testimonios incluían muestras de gratitud por compartir su experiencia, aspecto previamente identificado como una oportunidad para comunicar miedos o vivencias no deseadas^38^.

Para ampliar la capacidad de escucha a las madres, en algunos países se realizan encuestas masivas como es el caso de Austria^39^, Estados Unidos^40^ o Inglaterra^41^. Pero, desde el conocimiento de las autoras, este estudio es el primero que se realiza en España incorporando la influencia del diseño en la experiencia del parto. Una revisión sistemática reciente^42^ ha identificado artículos similares estudiados desde países como Estados Unidos^35^, Suecia^43^, Australia^44^, Irán^45^, China^46^, Canadá^47^, Dinamarca^37^, Reino Unido^48^ o Grecia^49^. Aun así, ninguno de ellos se basa en testimonios auto relatados por las madres que pueden influir en esos diseños.

Sin embargo, el estudio cuenta con distintas limitaciones. En cuanto a la muestra, los resultados no se han estratificado por variables sociodemográficas (como nacionalidad, edad o número de hijos), ya que se seleccionaron exclusivamente por su capacidad (formativa o profesional) para describir el entorno del parto en el hospital y el impacto en su experiencia. La selección de participantes a partir del muestreo por propósito complementado por bola de nieve, puede haber condicionado el relato final. Aunque tan solo dos de los testimonios recogidos se producen durante la pandemia, en ningún momento del relato se hace referencia a la emergencia sanitaria. Por otro lado, se desestiman los testimonios de partos en casa, porque no formaban parte del entorno analizado, que era el hospitalario. No obstante, la mayor limitación de esta investigación puede ser la antigüedad de los partos en el momento de ser contado ya que puede afectar a la veracidad de los hechos o a que se describan paritorios anticuados que ya hayan sido reformados. Sin embargo, se ha decidido mantener testimonios antiguos e incluso un testimonio con una antigüedad inferior a los 12 meses, por la exhaustividad de información aportada.

Precisamente por este último motivo, se plantean dos líneas de investigación. La primera sería analizar el estado actual de los bloques obstétricos en toda España, utilizando una encuesta que se podría elaborar a partir de una herramienta previamente preparada^50^, tal y como se está realizando para las unidades neonatales^51^. Una vez obtenido el diagnóstico actual del diseño arquitectónico, se podría continuar con una segunda línea de investigación para vincularla con resultados clínicos. Por ejemplo, relacionando los parámetros arquitectónicos de las unidades encuestadas con el buscador nacional de tasas de cesáreas por hospital^52^.

Como conclusión, el entorno del parto en el hospital debe ofrecer una primera impresión acogedora y personal para la parturienta, limitando los traslados innecesarios y reduciendo las distancias entre salas. Además, debe prestar atención al diseño de los pasillos y de sus techos, y posibilitar un contacto permanente con la naturaleza (a través de ventanas y jardines accesibles). Todas las habitaciones donde la madre pasa un tiempo prolongado deberían hacerla sentir arropada y acompañada durante el proceso, con espacio suficiente para su libre movimiento, con la capacidad de personalizar el entorno, con temperatura regulable, con espacio para la estancia de acompañantes, preservando su intimidad, descanso y decoro. No se puede descuidar el diseño del aseo, ni tampoco el quirófano debe permanecer inmutable ante una cesárea y el nacimiento de una nueva familia. Por otro lado, el diseño de la unidad de neonatos debería integrar a la familia para facilitar el apego con el bebé. En definitiva, son necesarios más estudios que incorporen la experiencia de la mujer en el proceso del parto, para promocionar políticas de diseño basadas en evidencias.
